# Selective Removal of Hemoglobin from Blood Using Hierarchical Copper Shells Anchored to Magnetic Nanoparticles

**DOI:** 10.1155/2017/7309481

**Published:** 2017-02-21

**Authors:** Youxun Liu, Yaokun Wang, Mingyang Yan, Juan Huang

**Affiliations:** ^1^School of Basic Medical Sciences, Xinxiang Medical University, Jinsui Avenue 601, Xinxiang, Henan 453003, China; ^2^Henan Collaborative Innovation Center of Molecular Diagnostics and Laboratory Medicine, Jinsui Avenue 601, Xinxiang, Henan 453003, China

## Abstract

Hierarchical copper shells anchored on magnetic nanoparticles were designed and fabricated to selectively deplete hemoglobin from human blood by immobilized metal affinity chromatography. Briefly, CoFe_2_O_4_ nanoparticles coated with polyacrylic acid were first synthesized by a one-pot solvothermal method. Hierarchical copper shells were then deposited by immobilizing Cu^2+^ on nanoparticles and subsequently by reducing between the solid CoFe_2_O_4_@COOH and copper solution with NaBH_4_. The resulting nanoparticles were characterized by scanning electron microscopy, transmission electron microscopy, Fourier transform infrared spectrometry, X-ray photoelectron spectroscopy, and vibrating sample magnetometry. The particles were also tested against purified bovine hemoglobin over a range of pH, contact time, and initial protein concentration. Hemoglobin adsorption followed pseudo-second-order kinetics and reached equilibrium in 90 min. Isothermal data also fit the Langmuir model well, with calculated maximum adsorption capacity 666 mg g^−1^. Due to the high density of Cu^2+^ on the shell, the nanoparticles efficiently and selectively deplete hemoglobin from human blood. Taken together, the results demonstrate that the particles with hierarchical copper shells effectively remove abundant, histidine-rich proteins, such as hemoglobin from human blood, and thereby minimize interference in diagnostic and other assays.

## 1. Introduction

In clinical diagnosis and medical research, changes in blood indices are critical parameters that reflect physiological status or indicate pathology [1]. However, some biomarkers in the blood, including DNA, RNA, and many proteins, are generally present at very low concentrations [2, 3] and are difficult to detect due to severe interference from highly abundant proteins such as hemoglobin [4, 5]. Consequently, depletion of such abundant proteins is an essential prerequisite.

Among the many depletion methods currently available, affinity-based techniques are some of the most effective and have become routine [6–8]. For example, immobilized metal affinity chromatography is now frequently used to separate histidine-rich proteins based on the selective interaction between immobilized metal ions and electron donor groups in proteins containing several consecutive histidine residues [9]. Separation is typically achieved via transition metal ions like Cu^2+^, Ni^2+^, Zn^2+^, or Co^2+^, which are typically chelated into molecules covalently bound to matrices such as dextrin gels, polystyrene beads, or nanoparticles. Of these, magnetic nanoparticles (MNPs) have been widely used because of biocompatibility, biodegradability, stability, facile surface functionalization, and magnetic features that facilitate convenient separation. Indeed, these nanoparticles have been used not only in protein purification, but also in drug delivery, diagnostic imaging, pollution removal, and enzyme immobilization [10–13]. For instance, metals immobilized on a magnetic matrix are widely used to effectively deplete abundant proteins and peptides from proteomic samples [14]. Unfortunately, the most commonly used MNPs are based on iron oxides and are thus susceptible to leaching in acidic conditions. Consequently, inorganic SiO_2_ has been used as a shell to protect magnetic cores [15, 16], although such a shell may also significantly reduce magnetism. Thus, it is necessary to fabricate new multifunctional composites that are both strongly magnetic and chemically stable. CoFe_2_O_4_, a thermally and chemically stable ferrite, appears to be one such composite [17, 18].

Cu^2+^ and Ni^2+^ are the most frequently used metal ions to magnetically separate proteins by affinity chromatography. For example, Chen et al. [19, 20] fabricated MNPs coated with iminodiacetic acid and charged with Cu^2+^ or Ni^2+^ to selectively adsorb histidine-rich bovine hemoglobin (BHb). BHb was also selectively captured on Cu^2+^-EDTA-Fe_3_O_4_ magnetic particles synthesized by Ding et al. through a one-pot solvothermal method [21]. However, a major disadvantage of the above systems is the low metal ion density on the surface, which leads to a low adsorption capacity for protein and restricts their practical application in protein separation. Consequently, well-designed magnetic nanomaterials with higher metal density have emerged as powerful candidates for meeting the above-mentioned requirement. Recently, Liu and coworkers [22] achieved highly selective isolation of polyhistidine-tagged proteins from cell lysates using a graphene-nickel composite. Intriguingly, Lee et al. [23] demonstrated that Ni nanoparticles are coated with NiO capture and magnetically separate histidine-tagged proteins. A hierarchical nickel shell anchored on silica coated magnetic Fe_3_O_4_@SiO_2_ nanoparticles has been designed and constructed, and the high Ni^2+^ density is found on the shell, which results in a high adsorption capacity for BHb [24]. The above literature shows that a hierarchical nickel shell can provide higher metal ion density on the surface of MNPs, which help to improve the protein adsorption capacity. However, there are few reports that Cu-coated core-shell magnetite nanoparticles are used for selective capture of histidine-rich proteins. This inspired us to evaluate whether magnetite nanoparticles coated with copper could be applied to selectively capture histidine-rich BHb or human hemoglobin. We note that Cu-coated core-shell CoFe_2_O_4_ composite microspheres are easily and magnetically separated from aqueous solutions and have improved chemical stability due to addition of Co^2+^. Further, Cu shell/core architecture may also protect a magnetic core without compromising purification efficiency. More importantly, a hierarchical copper shell may provide a high Cu^2+^ density on the surface of MNPs, which can increase adsorption capacity for protein.

Here, we describe a simple method to synthesize carboxyl-functionalized, Cu-coated, magnetic CoFe_2_O_4_ composite microspheres that rapidly and efficiently capture proteins like hemoglobin. In this method, CoFe_2_O_4_ particles functionalized with polyacrylic acid are first synthesized via a one-pot solvothermal route and then coated with copper ions. The copper ions are then reduced to Cu metal by aqueous NaBH_4_, resulting in anchored, hierarchical Cu shells. The particles were then characterized without further processing by scanning electron microscopy (SEM), transmission electron microscopy (TEM), Fourier transform infrared spectrometry (FT-IR), energy dispersive X-ray spectroscopy (EDX), and vibrating sample magnetometry (VSM). The particles were then tested against bovine hemoglobin to assess kinetic and isothermal adsorption. Finally, the particles were tested against human blood.

## 2. Materials and Methods

### 2.1. Reagents and Materials

All chemicals, including ferric chloride hexahydrate (FeCl_3_·6H_2_O), cobalt chloride (CoCl_2_), copper (II) sulfate pentahydrate (CuSO_4_·5H_2_O), polyacrylic acid, and sodium borohydride (NaBH_4_) were analytical grade (Sinopharm Chemical Reagent Company) and were used as received without further purification. Deionized water was used throughout. Protein molecular weight markers, bovine hemoglobin (BHb, molecular weight 64.5 kDa, pI 6.8), and bovine serum albumin (BSA, molecular weight 67 kDa, pI 4.7) were purchased from Dingguo Biological Technology Company (Beijing, China). Human blood was obtained from a healthy volunteer and immediately used.

### 2.2. Synthesis of Cu Magnetic Particles

Cu magnetic particles (Cu-MNPs) were synthesized in two continuous steps. First, magnetic particles functionalized with polyacrylic acid were synthesized via a simple solvothermal route as previously described [24], with some modification. Briefly, FeCl_3_·6H_2_O (0.5 g) and CoCl_2_ (0.23 g) were dissolved into a clear solution in 50 mL water and mixed with 15 mL polyacrylic acid (PAA) and 15 mL 80% hydrated hydrazine. The mixture was vigorously stirred until homogeneous, transferred to a 250 mL Teflon-lined stainless-steel autoclave, and reacted at 180°C for 10 h. The final product was collected with a magnet, washed with ethanol and deionized water several times in this order, and dried overnight at 50°C in a vacuum oven. The PAA@MNPs were obtained. Subsequently, 0.5 g of the product was mixed with 50 mL 0.2 mM CuSO_4_ and then with 10 mL water containing 0.4 g NaBH_4_. The mixture was mechanically stirred at room temperature overnight, and the resulting nanoparticles were isolated by magnetic decantation, washed with ethanol and deionized water several times in this order, and dried overnight at 50°C in a vacuum oven. Following the steps above, Co^2+^@PAA@MNPs, Ni^2+^@PAA@MNPs, and Cu^2+^ @PAA@MNPs were synthesized without adding NaBH4.

### 2.3. Characterization

Nanoparticles were characterized on an FT-IR spectrometer (Thermo Nicolet Corporation, Waltham, MA, USA), a Zetasizer Nano 100 (Malvern, Worcestershire, UK), and a high-resolution transmission electron microscope (HRTEM; JEOL-2010, Japan). SEM and EDX micrographs were obtained with a field emission scanning electron microscope (ZEISS Ultra 55, Carl Zeiss, Germany), while X-ray photoelectron spectra were recorded on an electron spectrometer emitting a 300 W Al KR radiation (ESCALAB220i-XL, VG Scientific, UK). Magnetization was measured at room temperature on a vibrating sample magnetometer (PPMS-9, Quantum Design, San Diego, USA).

### 2.4. Adsorption of BHb

**Figure 1:**
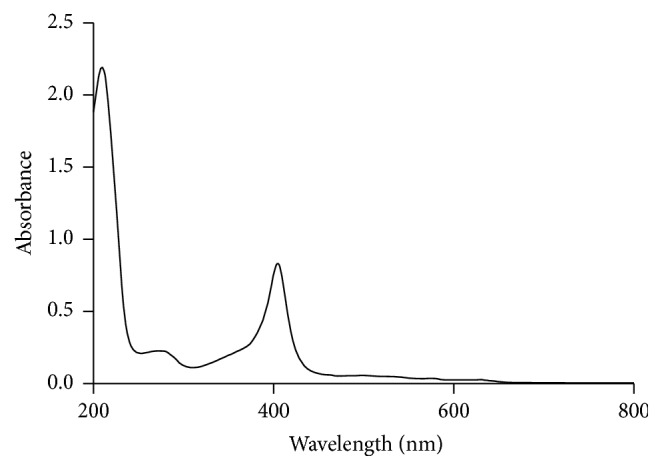
UV-visible spectrum of BHb.

Due to its similarity to human hemoglobin, BHb was used as a model histidine-rich protein. Adsorption to Cu-MNPs was assessed over a range of pH, contact time, and initial protein concentration. The adsorption kinetic and adsorption isotherm were measured in batch experiments. In a typical adsorption experiment, 0.02 g Cu-MNPs was shaken for 90 min at room temperature with 20 mL 1 mg/mL BHb in Tris-HCl pH 8.5. After magnetic decantation, the absorbance of the supernatant at 408 nm was measured using a UV-1700 spectrophotometer (Shimadzu, Kyoto, Japan). We note that BHb has maximum absorbance at 408 nm (Figure 1).

Adsorption was calculated according to

(1) $$\begin{array}{c} Q=\frac{\left(C_{0}-C_{1}\right)V}{M}, \end{array}$$

where *Q* (mg g^−1^) is the amount of BHb adsorbed, *C*_0_ (mg mL^−1^), and *C*_1_ (mg mL^−1^) are the initial and residual concentration of BHb in the supernatant, respectively, *V* (mL) is the reaction volume, and *M* (g) is the mass of Cu-MNPs. The effect of pH on adsorption was tested from 4.0 to 7.0 in 0.1 M citric acid sodium hydrogen phosphate buffer, and from 7.5 to 9.5 in 0.1 M Tris-HCl buffer. The effect of contact time was assessed while keeping the initial concentration of BHb constant at 1.0 mg mL^−1^. Adsorption kinetic data were fitted to pseudo-first-order or pseudo-second-order models [25, 26], which can be expressed as

(2) ln⁡Qe−Qt=ln⁡Qe−k1t

(3) tQt=1K2Qe2+tQe,

where *Q*_*t*_ (mg g^−1^) is the adsorbed BHb at time *t* and and *Q*_*e*_ (mg g^−1^) is the adsorbed BHb at equilibrium. *k*_1_ (min^−1^) and *k*_2_ (g mg^−1^ min^−1^) are the pseudo-first-order rate constant and pseudo-second-order rate constant, respectively.

The effect of initial protein concentration was investigated from 0.1 to 3.0 mg mL^−1^, and the adsorption isothermal data were fitted to the Freundlich model or the Langmuir [27, 28], which can be expressed as

(4) CeQe=CeQm+1KLQm

(5) ln⁡Qe=ln⁡KF+1nln⁡Ce,

where *Q*_*e*_ (mg g^−1^) is the adsorbed BHb at equilibrium and *Q*_*m*_ (mg g^−1^) is the maximum BHb that can be adsorbed. *C*_*e*_ (mg mL^−1^) denotes the residual concentration of BHb in solution at equilibrium, while *K*_*F*_ (mg g^−1^) is the Freundlich constant reflecting the maximum adsorption capacity. 1/*n* is the adsorption intensity and *K*_*L*_ (mL mg^−1^) is the Langmuir constant, which is related to the energy of adsorption.

### 2.5. Selectivity

Due to similarities in abundance, molecular weight, and volume [29], BSA was used as a model competing protein to evaluate the selectivity of Cu-MNPs for BHb. Selectivity was tested using 20 mL solutions containing either protein at 1 mg mL^−1^ or a mix of both proteins at 1 mg mL^−1^ each. Supernatants were analyzed on a UV-1700 spectrophotometer (Shimadzu, Kyoto, Japan), as BHb and serum albumin have characteristic absorbance peaks at 406 nm and 280 nm, respectively.

### 2.6. Depletion of Hemoglobin from Human Blood

To evaluate potential use in the clinic, Cu-MNPs (0.02 g) were used to deplete hemoglobin from a sample of human blood, which was diluted 100-fold in 0.1 M Tris-HCl pH 8.5. After magnetic separation, depleted and control samples were analyzed by spectrophotometry and sodium dodecyl sulfate-polyacrylamide gel electrophoresis (SDS-PAGE).

## 3. Results and Discussion

### 3.1. Adsorption Capacity of the Nanoparticles with Different Metal Ions on the Surface

**Figure 2:**
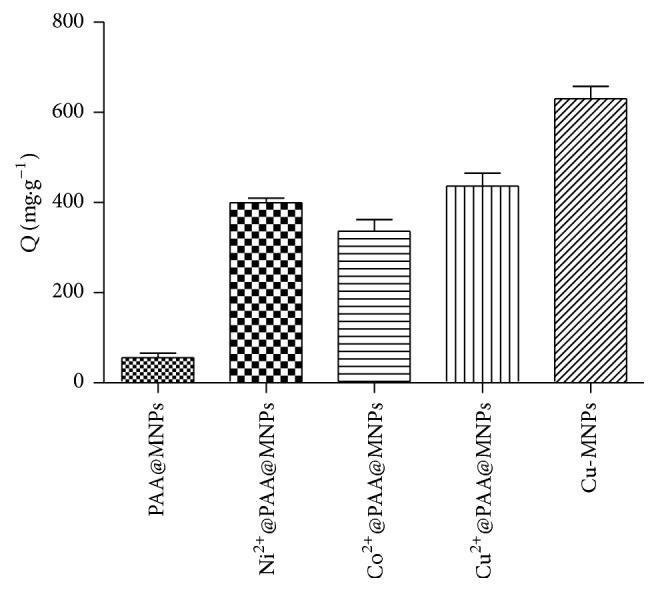
Adsorption capacity of the nanoparticles with different metal ions on the surface.

As shown in Figure 2, the adsorption capacities of the PAA@MNPs, Co^2+^@PAA@MNPs, Ni^2+^@PAA@MNPs, and Cu^2+^@PAA@MNPs were compared. The result showed that the adsorption capacity of MNPs with Cu^2+^ on the surface was highest among these nanoparticles, so Cu^2+^ was chosen for BHb adsorption and magnetic particles with copper shell had been explored further for BHb adsorption. In fact, the adsorption capacity of MNPs after coating copper shell was higher than that of Cu^2+^ @PAA@MNPs.

### 3.2. Synthesis and Characterization of Cu-MNPs

**Scheme 1:**
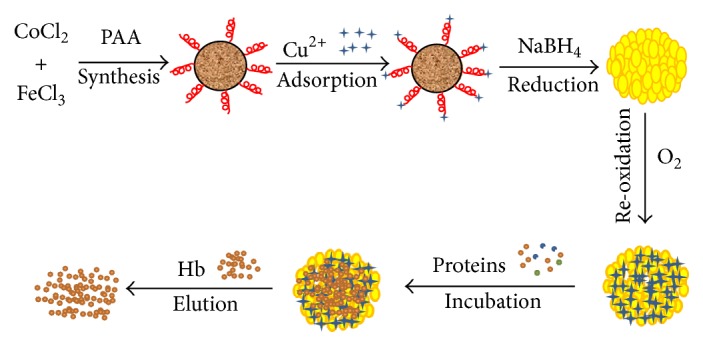
Schematic illustration of the strategy for preparation of Cu-MNPs and Hb adsorption.

**Figure 3:**
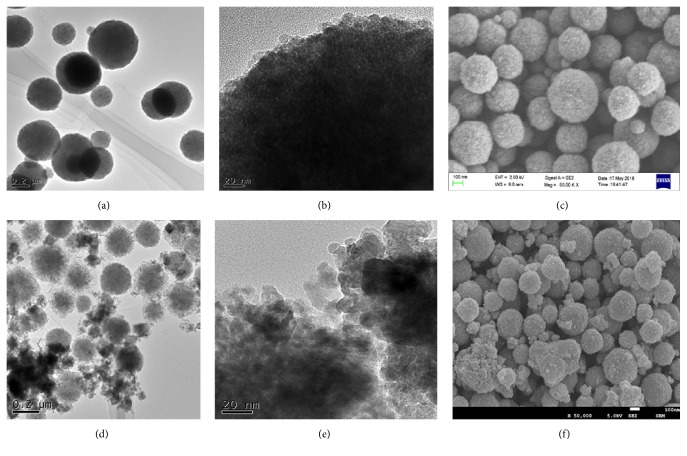
TEM images of magnetic nanoparticles (P-MNPs) before (a, b) and after loading with copper (Cu-MNPs) (d, e). SEM images of magnetic nanoparticles (P-MNPs) before (c) and after loading with copper (Cu-MNPs) (f).

The preparation of Cu-MNPs is illustrated in Scheme 1, along with the proposed mechanism by which it separates target proteins by metal affinity. Surface morphology and architecture are shown in TEM and SEM images in Figure 3, which reveal that magnetic nanoparticles functionalized with polyacrylic acid (P-MNPs) are spherical before (Figure 3(a)) and after loading with Cu (Figure 3(d)). Based on TEM images, the nanoparticles gained an obvious flower-like core with a clear shell upon loading with copper (Figure 3(d)). In comparison to P-MNPs (Figures 3(b) and 3(c)), the surface became rougher and very fine particles appeared (Figures 3(e) and 3(f)) after immobilized Cu^2+^ was reduced to Cu metal by NaBH_4_, perhaps due to the formation of Cu core/shell composite microspheres. The TEM images showed that P-MNPs were well-dispersed, but Cu-MNPs had slight aggregation in water. Furthermore, these TEM and SEM images indicated that the sizes of P-MNPs and Cu-MNPs were not uniform.

**Figure 4:**
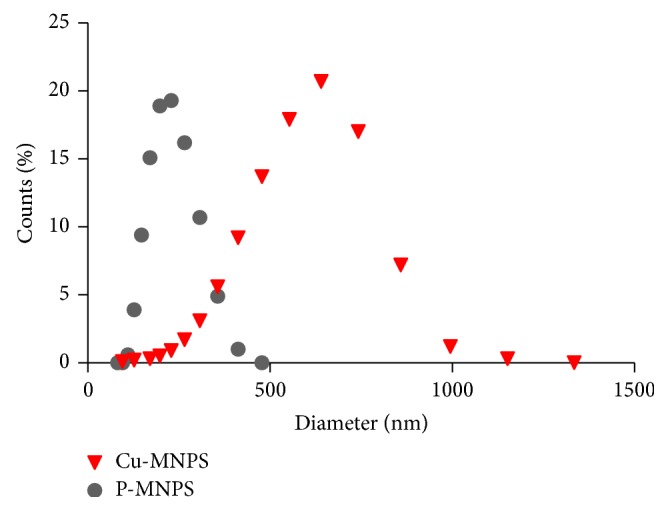
Particle size distribution of magnetic nanoparticles with or without copper.

Figure 4 shows the particle size distribution of magnetic nanoparticles with or without copper. The latter had an average hydrodynamic size of about 281 nm, the hydrodynamic diameter increased to 513 nm after loading, indicating that a very thick Cu shell may have been deposited.

**Figure 5:**
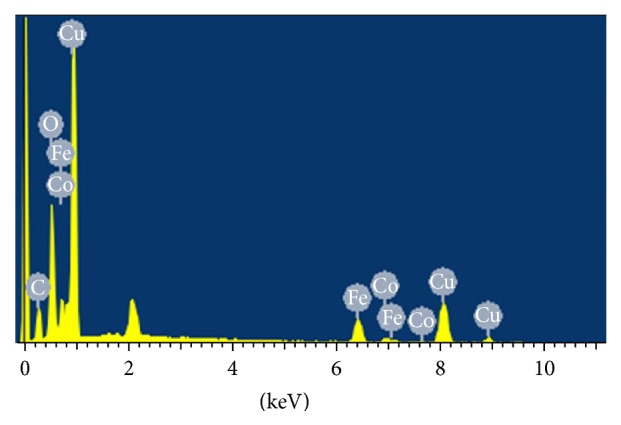
Energy dispersive X-ray spectrum of Cu-MNPs.

Copper is easily identified in the energy dispersive X-ray spectra of selected surfaces in Cu-MNPs (Figure 5). Remarkably, the particles are more than 40% Cu by weight (Table 1), implying that a large amount of the metal was immobilized, and corroborating the previous result that copper-loaded nanoparticles are larger than unloaded particles.

**Figure 6:**
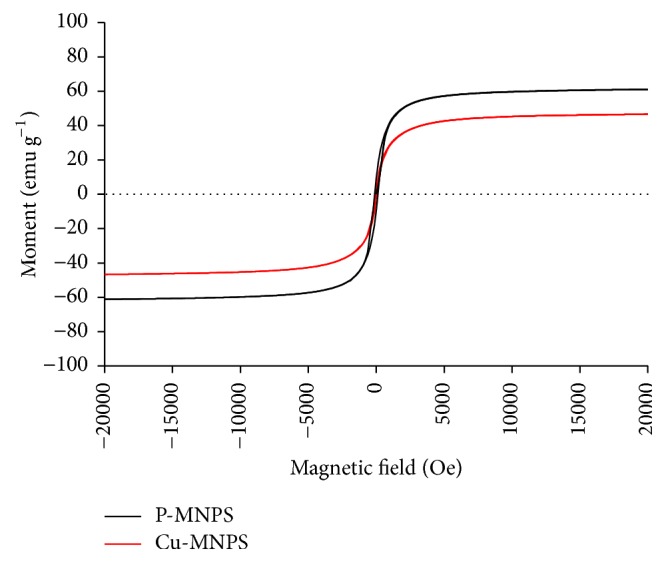
Vibrating sample magnetometry curves of u P-MNPs and Cu-MNPs.

Vibrating sample magnetometry at room temperature indicated that the saturation magnetization value was 60 emu g^−1^ and 48.0 emu g^−1^ for unloaded and copper-loaded magnetic nanoparticles, respectively (Figure 6). Although magnetization slightly decreased after copper loading, the remaining magnetic strength was sufficient to allow easy separation of copper-loaded particles from aqueous solutions in less than 30 s by applying an external magnetic field.

**Figure 7:**
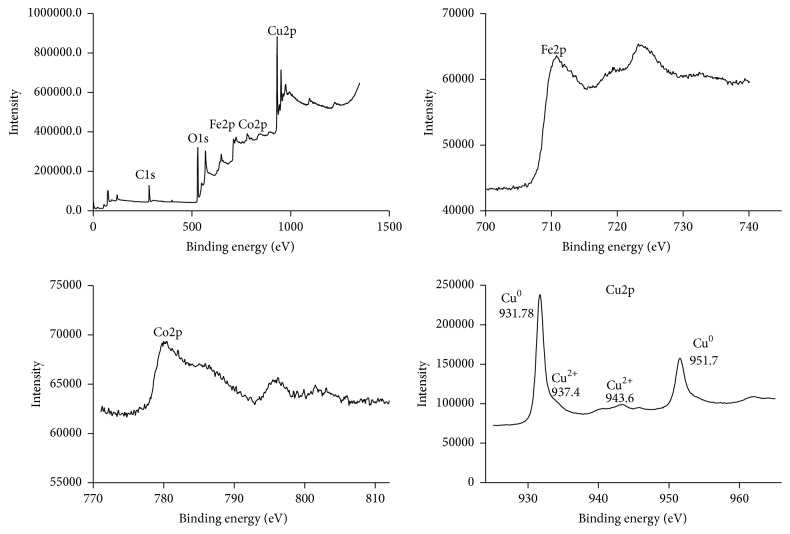
XPS spectra of the Cu-MNPs.

The survey and the respective element XPS of Cu-MNPs are given in Figure 7. The binding energies of O (1s, 529.88 eV), C (1s, 284.07 eV), Fe (2p, 710.3 eV), Co (2p, 781.09 eV), and Cu (2p, 931.78 eV) are obviously observed. XPS shows that the oxidation state of copper was a mixture of oxidized forms including two states (Cu^0^, Cu^1+^ and Cu^2+^). The strong peak at 931.78 can be ascribed to Cu^0^ and/or Cu^1+^ while the small peak around 937.6 can be ascribed to Cu^2+^. It has been reported that Cu^0^ and Cu^1+^ are hard to distinguish owing to that there is about 0.3 eV between them in binding energy [30]. The small peak at 943.6 reveals a Cu^2+^ oxidized form. In addition, the presence of the dominant peak at 951.7 can be assigned to Cu^0^ and/or Cu^1+^. The proportion of Cu^0^ and/or Cu^1+^: Cu^2+^ can be deduced to be about 8 : 1 by integrating the area in the XPS. Based on above results shown in XPS, it can therefore be reasonably concluded that the deposition of metallic Cu^0^ species on the magnetic nanoparticle surface occurred due to the reduction of Cu^2+^ to metallic Cu^0^ by NaBH_4_. On the other hand, the existence of Cu^1+^ and Cu^2+^ species on the magnetic nanoparticle surface was due to the fact that many metallic Cu^0^ species subsequently were reoxidized back Cu^1+^ and Cu^2+^ species in an aqueous environment resulting in a mixture of oxidized forms. A similar phenomenon was observed by Wang and colleagues [31]. Importantly, a high density of Cu^2+^on the magnetic nanoparticle surface will contribute to protein adsorption by Cu^2+^-chelate affinity chromatography.

**Figure 8:**
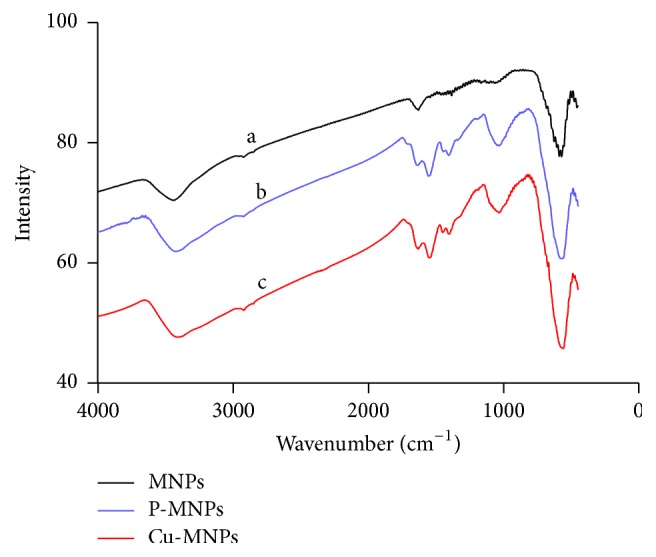
FT-IR spectra of MNPs, P-MNPs, and Cu-MNPs.

In FT-IR spectra of magnetic nanoparticles coated with or without polyacrylic acid and copper (Figure 8), the strong IR band at 580 cm^−1^ is attributed to Fe-O vibrations, whereas bands at 2927, 1032, and 1647 cm^−1^ are associated with C-H stretching, aliphatic C-N stretching, and C-O stretching vibrations due to polyacrylic acid, respectively (Figure 8, curves a, b, and c) [24, 30], confirming that the nanoparticles were successfully functionalized with polyacrylic acid during synthesis.

### 3.3. The Stability of Cu-MNPs

**Figure 9:**
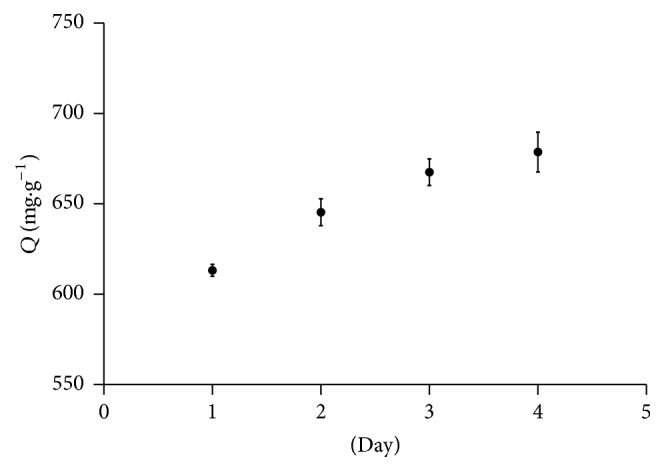
The amount of BHb adsorbed to Cu-MNPs as a function of time.

The amount of BHb adsorbed to Cu-MNPs as a function of time was measured to evaluate the stability of copper shell. As shown in Figure 9, the amount of BHb adsorbed to Cu-MNPs gradually increased with day, indicating that the content of Cu^1+^or Cu^2+^ on copper shell increased, which was attributed to the continuing oxidation of copper shell. This phenomenon would result in two different effects. On the one hand, an increased density of Cu^1+^or Cu^2+^ may contribute to protein adsorption. On the other hand, the continuing oxidation of copper shell would reduce stability of Cu-MNPs.

### 3.4. Effect of pH

**Figure 10:**
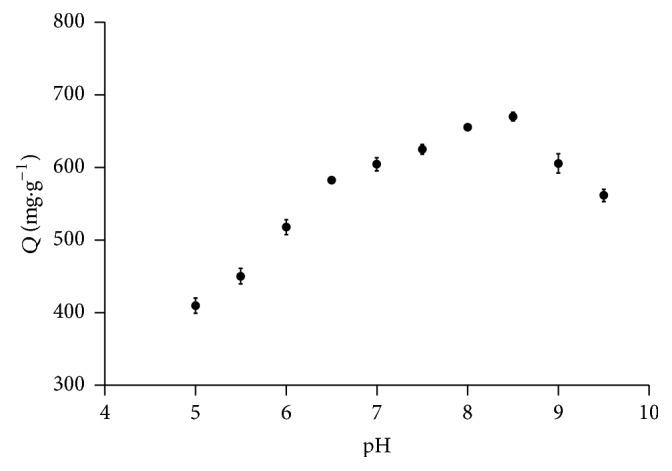
Effect of pH on adsorption of BHb to Cu-MNPs.

As can be seen in Figure 10, adsorption of BHb to Cu-MNPs was strongly pH-dependent, with maximum adsorption at about pH 8.5. Adsorption decreased below and above pH 8.5, in line with previous studies indicating that the optimal pH is between 8.0 and 9.0 [21]. With an isoelectric point near pH 7.0, BHb should be negatively charged above pH 7.0 [31]. This was in favor of BHb adsorption by the as-synthesized nanoparticles which carried a lot of positive charges due to the existence of a large amount of Cu^2+^ and Cu^1+^ on the surface. The pH of the buffer has profound effect on the adsorption process owing that it can affect the charge of protein and the surface charge status of adsorbents [32, 33]. Accordingly, buffers at pH 8.5 were used in all subsequent experiments to preserve the electrostatic status of the target protein and the nanoparticles.

### 3.5. Effect of Contact Time and Adsorption Kinetics

**Figure 11:**
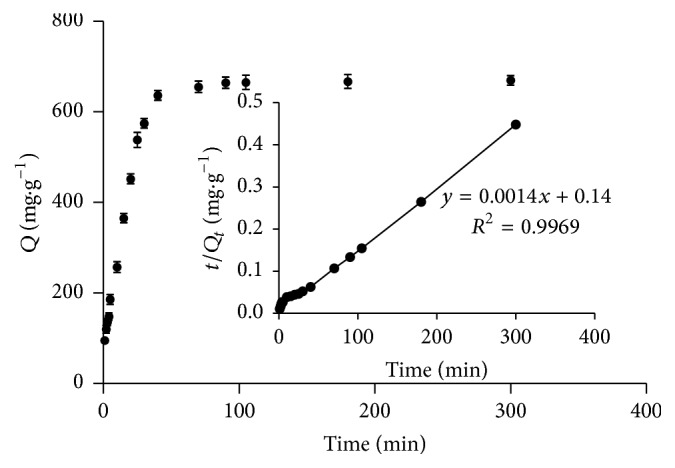
Time course of BHb adsorption to Cu-MNPs.

Protein adsorption is widely interpreted in terms of well-established kinetic models [25]. As illustrated in Figure 11, adsorption of BHb to copper magnetic particles sharply increased from 0 to 30 min due to a large number of available sorption sites. However, adsorption tapered off thereafter and reached equilibrium in 90 min. Accordingly, all subsequent adsorption experiments were allowed to proceed for 90 min. Kinetic data were then fit to pseudo-first-order and pseudo-second-order kinetic models. The data fit to a pseudo-first-order model reasonably well, with equation (2) ln⁡(674.2 − *Q*_*t*_) = 6.8431 − 0.0917*t* and determination coefficient (*R*^2^) 0.9727. On the other hand, the data fit significantly better to a pseudo-second-order model with equation (3) *t*/*Qt* = 0.014*t* + 0.133 and determination coefficient 0.9976.

### 3.6. Effect of Initial Protein Concentration and Isothermal Adsorption

**Figure 12:**
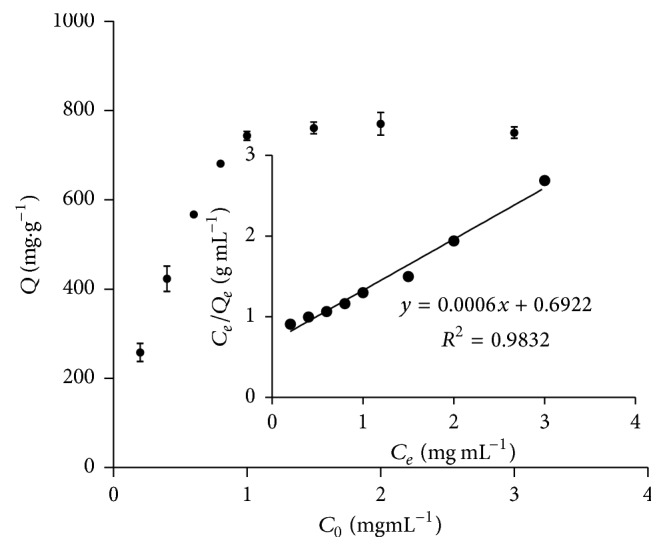
Effect of initial protein concentration on the amount of BHb adsorbed to Cu-MNPs in 90 min.

Figure 12 shows the effects of initial protein concentration on the amount of BHb adsorbed to Cu-MNPs in 90 min. Adsorption increased with initial protein concentration up to 1.0 mg mL^−1^. Accordingly, the adsorption capacity for BHb was determined to be 670 mg g^−1^, which is higher than previously reported for similar devices [34, 35]. Presumably, this is due to the extremely high density of immobilized Cu^2+^ ions. We note that adsorption of proteins to Cu-MNPs is attributable to metal affinity, as well as to electrostatic and hydrophobic interactions. Indeed, immobilized Cu^2+^ ions in nanoparticles interact strongly with protein electron donor groups such as histidine residues. In addition, proteins like BHb are negatively charged in basic aqueous solutions and thus can electrostatically interact with positively charged bodies like Cu-MNPs [21].

Experimental isothermal data were fit to the Langmuir and Freundlich models as shown in Figure 12. Fitting to the Langmuir model yielded the equation (4) *C*_*e*_/*Q*_*e*_ = 0.0015*C*_*e*_ + 0.1712, with determination coefficient 0.9986. Based on this equation, the maximum adsorption capacity (*Q*_*m*_) was calculated to be 666 mg g^−1^, which is very close to the experimental value of about 670 mg g^−1^. On the other hand, fitting to the Freundlich model yielded equation (5); a significantly lower determination coefficient (*R*^2^) of 0.8889 was obtained. Thus, isothermal data fit the Langmuir model significantly better, indicating that BHb interacts with Cu-MNPs by monolayer adsorption [21].

### 3.7. Selectivity

**Figure 13:**
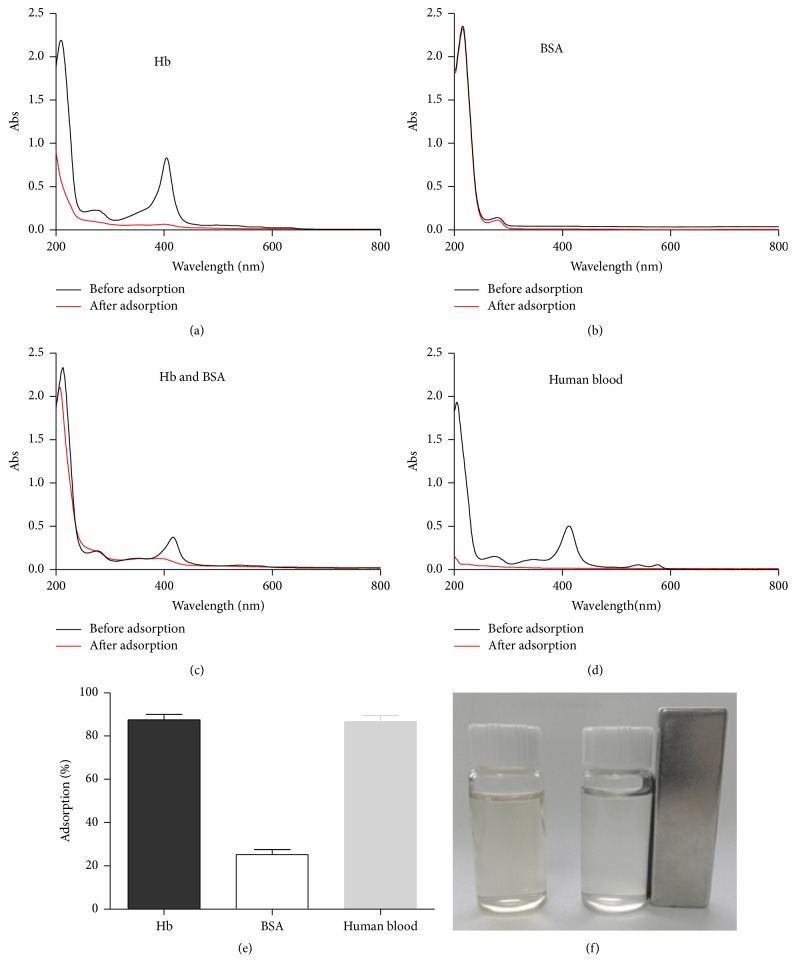
Selectivity of Cu-MNPs for BHb, as assessed visually (f) and by UV-Visible spectrophotometry.

Due to similarities in abundance, molecular weight, and volume [36], BSA was used as a model competitor to evaluate the selectivity of Cu-MNPs for BHb. BSA and hemoglobin produce characteristic peaks at 280 nm and 406 nm in the UV-visible range (Figure 13), respectively. Thus, these peaks were also used to estimate their respective concentrations. The peak at 406 nm sharply decreased and nearly disappeared from the supernatant after BHb was incubated with Cu-MNPs by itself (Figure 13(a)), indicating efficient and selective adsorption. In contrast, the peak at 280 nm did not significantly decrease after adsorption of solutions containing BSA, by itself or in combination with BHb (Figures 13(b) and 13(c)), indicating negligible adsorption. Accordingly, the adsorption rate was calculated to be 87% for BHb and 25% for BSA (Figure 13(e)). In addition, solutions containing BHb, by itself or in combination with BSA, turned from reddish brown to a light color after incubation with Cu-MNPs (Figure 13(f)), indicating specific adsorption of hemoglobin. Taken together, these results show that Cu-MNPs selectively adsorb BHb, likely because it contains 24 surface histidine residues, while BSA contains only two accessible histidines [37, 38]. Thus, BHb is much easier to capture than albumin by affinity to immobilized metal ions.

### 3.8. Depletion of Hemoglobin from Human Blood Samples

**Figure 14:**
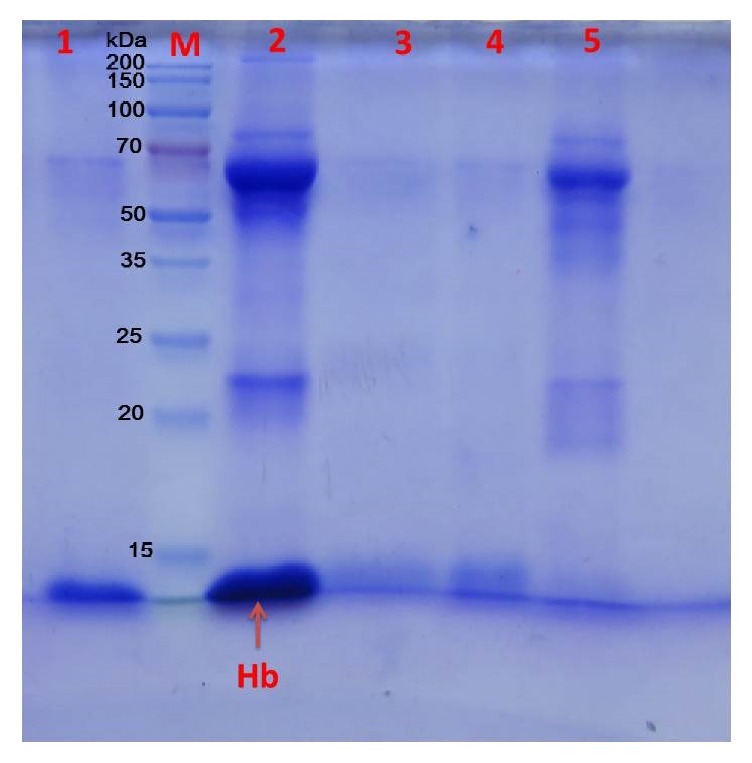
SDS-PAGE analysis of a human blood sample incubated with Cu-MNPs. Lane M, protein marker; lane 1, elution with 100 mM imidazole; lane 2, human blood diluted 100-fold; lane 3, elution with 10 mM imidazole; lane 4, elution with 20 mM imidazole; lane 5, supernatant after adsorption.

To assess potential utility in the clinic, a fresh sample of human blood was diluted 100-fold and incubated with Cu-MNPs. The characteristic hemoglobin peak at 406 nm clearly diminished after adsorption, along with the red color of the sample. Indeed, about 87% of hemoglobin was removed (Figure 13(e)). SDS-PAGE analysis (Figure 14) also indicated that the nanoparticles adsorbed hemoglobin, but not other proteins. Consequently, hemoglobin was recovered from nanoparticles after elution with 10, 20, and 100 mM imidazole (lanes 1, 3, and 4 in Figure 14). Collectively, the data indicate that Cu-MNPs may be used to effectively remove target proteins and have potential value in clinical testing of human blood.

## 4. Conclusions

In conclusion, we present a facile route to deposit hierarchical copper shells on magnetic nanoparticles, which can then be used to capture hemoglobin. We note that after deposition, copper shells undergo partial reoxidation to Cu^2+^ and Cu^1+^, resulting in a mixture of copper oxidized forms. This mixture both protects the magnetic core and provides a high density of Cu^2+^ ions that strongly adsorb histidine-rich proteins. Indeed, UV-visible spectrophotometry and SDS-PAGE analysis demonstrated that Cu-MNPs specifically capture BHb due to strong interactions between Cu^2+^ and histidine residues. Kinetic adsorption data followed pseudo-second-order kinetics, while isothermal data were described well by the Langmuir model. Importantly, the nanoparticles specifically depleted hemoglobin from human blood, demonstrating that the nanocomposites have potential application in removing highly abundant histidine-rich proteins from complex samples.

## Figures and Tables

**Table 1:** Elemental analysis of Cu-MNPs by energy-dispersive X-ray spectroscopy.

Element	Mass percentage (%)
C	21.49
N	0.51
O	19.74
Fe	10.89
Co	2.61
Cu	44.75

Total	100
